# Recurring episodes of bundle branch reentry ventricular tachycardia due to aortitis preceded by SARS-CoV-2 infection: a case report

**DOI:** 10.1186/s12872-023-03080-7

**Published:** 2023-01-25

**Authors:** Simon E. Melchior, Mikkel M. Schoos, Uffe Gang, Peter K. Jacobsen, Lothar Wiese, Thomas Maria Melchior

**Affiliations:** 1 Department of Cardiology, Bispebjerg and Frederiksberg Hospital, 2400 Copenhagen, Denmark; 2 Department of Cardiology, Zealand University Hospital, 4000 Roskilde, Denmark; 3 Department of Cardiology, Rigshospitalet, 2100 Copenhagen, Denmark; 4 Department of Infectious Disease, Zealand University Hospital, 4000 Roskilde, Denmark

**Keywords:** COVID-19, Aortitis, Bundle branch reentry ventricular tachycardia, VT ablation, MIS-A, Case report

## Abstract

**Background:**

SARS-CoV-2 may trigger both vasculitis and arrhythmias as part of a multisystem inflammatory syndrome described in children as well as in adults following COVID-19 infection with only minor respiratory symptoms. The syndrome denotes a severe dysfunction of one or more extra-pulmonary organ systems, with symptom onset approximately 2–5 weeks after the COVID-19 infection. In the present case, a seemingly intractable ventricular tachycardia preceded by SARS-CoV2 infection was only managed following the diagnosis and management of aortitis.

**Case presentation:**

A 69-year-old woman was hospitalized due to syncope, following a mild COVID-19 infection. She presented with paroxysmal atrial fibrillation and intermittent ventricular tachycardia interpreted as a septum-triggered bundle branch reentry ventricular tachycardia, unaffected by amiodaron, lidocaine and adenosine. A CT-scan revealed inflammation of the aortic arch, extending into the aortic root. In the following days, the tachycardia progressed to ventricular storm with intermittent third-degree AV block. A temporary pacemaker was implanted, and radiofrequency ablation was performed to both sides of the ventricular septum after which the ventricular tachycardia was non-inducible. Following supplemental prednisolone treatment, cardiac symptoms and arrythmia subsided, but recurred after tapering. Long-term prednisolone treatment was therefore initiated with no relapse in the following 14 months.

**Conclusion:**

We present a rare case of aortitis complicated with life-threatening ventricular tachycardia presided by Covid-19 infection without major respiratory symptoms. Given a known normal AV conduction prior to the COVID-19 infection, it seems likely that the ensuing aortitis in turn affected the septal myocardium, enabling the reentry tachycardia. Generally, bundle branch reentry tachycardia is best treated with radiofrequency ablation, but if it is due to aortitis with myocardial affection, long-term anti-inflammatory treatment is mandatory to prevent relapse and assure arrhythmia control. Our case highlights importance to recognize the existence of the multisystem inflammatory syndrome in adults (MIS-A) following COVID-19 infection in patients with alarming cardiovascular symptoms. The case shows that the early use of an CT-scan was crucial for both proper diagnosis and treatment option.

## Background

Most people who contract SARS-CoV-2 experience mild to moderate symptoms. In a few patients, however, the affection is more severe, especially in older men with known lung and/or cardio-vascular disease. Here, the respiratory symptoms predominate due to widespread lung infection that at worst can progress to multi-organ failure and death. As with any viral disease, the heart is oftentimes affected by a COVID-19 infection and thus elevated levels of high-sensitive troponin I have been detected in most patients admitted with COVID-19 [1, 2]. Fulminant myocarditis, on the other hand, is very rare. Involvement of the thoracic aortic wall and especially the aortic arch has been observed in some patients with COVID-19, but recurrent cardiac arrhythmias have not been reported in these patients [3–7].

Here we present the case of a patient admitted to hospital with life-threatening ventricular arrhythmia complicated by the appearance of a third-degree AV-block, who two months prior to admission had experienced a mild episode of COVID-19 documented by a PCR test with loss of appetite, general discomfort, and fatigue, but without fever or respiratory impairment.

## Case presentation

**Fig. 1:**
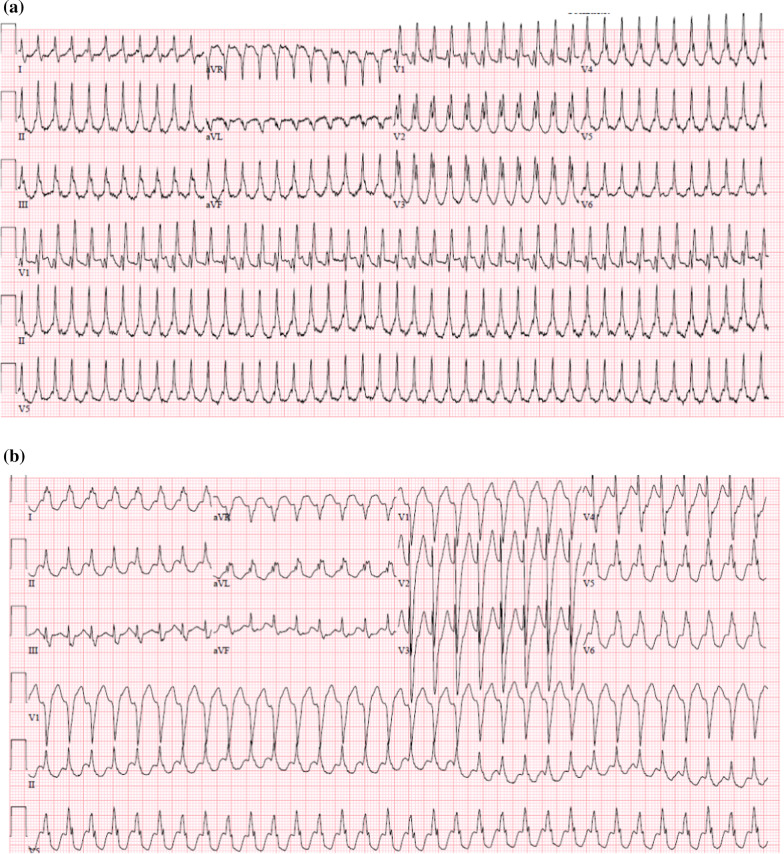
The ECG showing the ventricular tachycardia exhibiting a RBBB (**a**) or LBBB pattern (**b**)

A 69-year-old Caucasian woman was hospitalized after two episodes with syncope. She had a history of mild COPD and paroxysmal AFib treated with a beta-blocker. Two months prior to admission she had experienced a mild episode of COVID-19 without fever or respiratory impairment, documented by a PCR test. For weeks, she had experienced increasing dyspnea, frequent bouts of palpitations, dizziness, and near-syncope. Upon admittance, she complained of pain from the left thoracic wall after a fall at home. Clinically, she was affected by pain due to rib-fracture. She had no fewer and was hemodynamically stable despite a wide QRS complex tachycardia with a ventricular rate of 199 bpm. (Fig. 1a). The blood tests showed hemoglobin 6.1 mmol/L, leucocytes 12.9 10^9^/L, CRP 75 mg/L. S-potassium, s-magnesium, and troponin I were all within normal ranges. A Chest X-ray were inconspicuous and a throat swab for SARS-CoV-2 was negative. An echocardiogram demonstrated slightly reduced left ventricular function (LVEF 45%) and a mild mitral insufficiency. The tachycardia was initially treated with amiodarone.

**Fig. 2:**
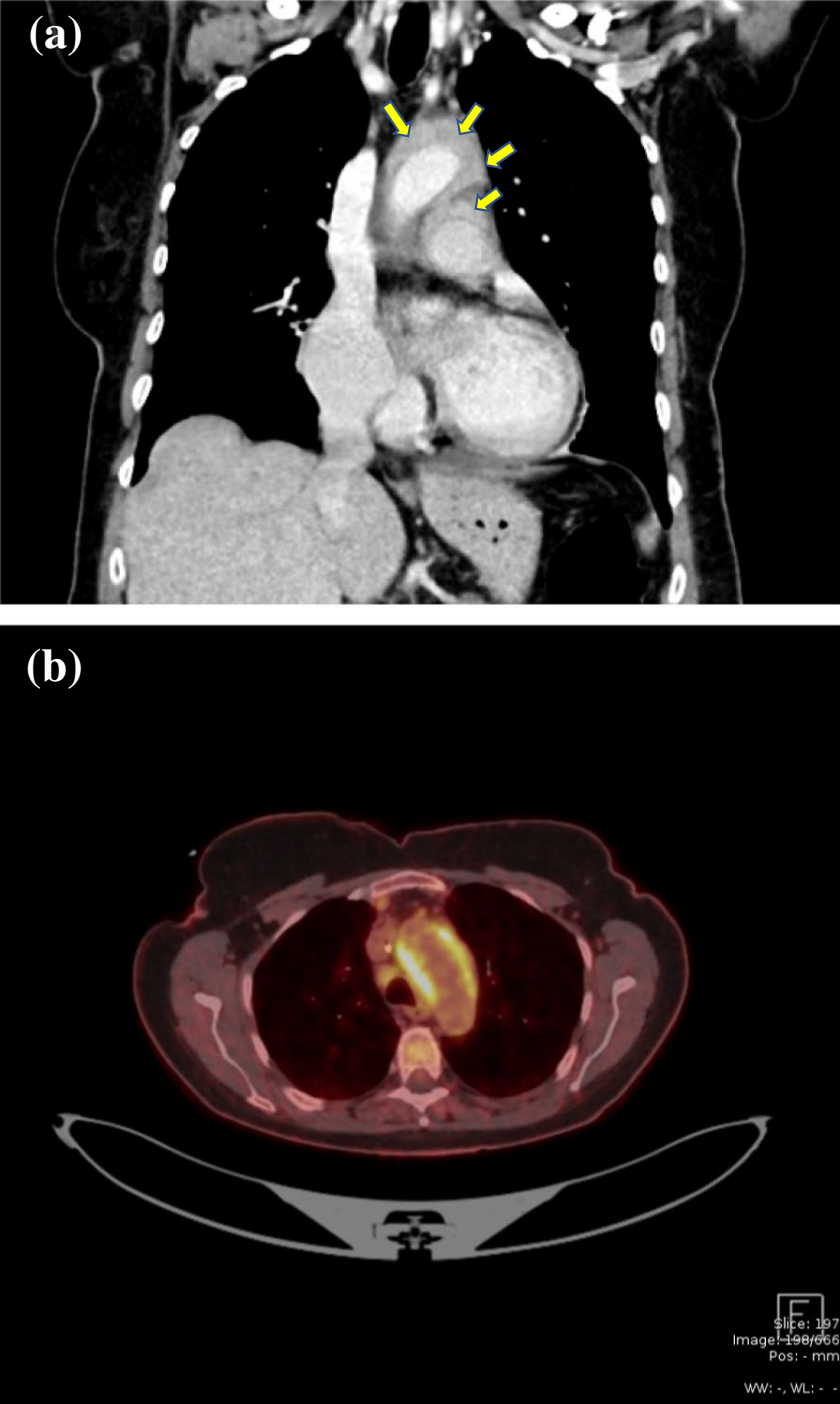
CT scan showing a markedly thickened aortic wall at the level of the aortic arch (1 cm) involving the wall of the pulmonary artery (**a**). PET scan showing the inflammation of the aortic arch (**b**)

An acute thoraco-abdominal CT-scan revealed not only several non-dislocated rib-fractures, but also a markedly thickened aortic wall (1 cm) at the level of the aortic arch. The wall thickening extended up along the left subclavian artery to the left carotid artery, and down into the aortic root. These findings were compatible with the diagnosis of aortitis (Fig. 2a). The CT-scan also ruled out aortic dilatation, dissection, aneurysms or stenotic segments. The consultant rheumatologist did not find any subjective signs of systemic involvement and thus suggested a whole-body PET scan, which revealed extensive areas of increased activity throughout the aortic root, ascending aorta, the pulmonary trunk as well as in the surrounding mediastinum (Fig. 2b). As systemic vasculitis as well as Giant cell arteritis were ruled out, a suspicion of SARS CoV-2-triggered vasculitis/perivasculitis involving the ascending aorta and arch lead to a reevaluation of the patient’s medical history. Antibody testing confirmed previous SARS-CoV-2 infection, whereas tests for HIV, tuberculosis and syphilis were negative.

The basic rhythm was AFib and the intermittent tachycardia was interpreted as being a high-septum-triggered bundle branch reentry ventricular tachycardia (BBRVT) unaffected by adenosine administration. The patient underwent an EP-study. The measured AV conduction proved to be poor despite isoprenaline infusion, but no arrhythmia was triggered, nor any accessory pathways. As the patient also started developing intermittent third-degree AV block, a temporary pacemaker was implanted. Antibiotics given empirically did not cause any reduction in CRP levels and blood cultures were all negative.

**Fig. 3:**
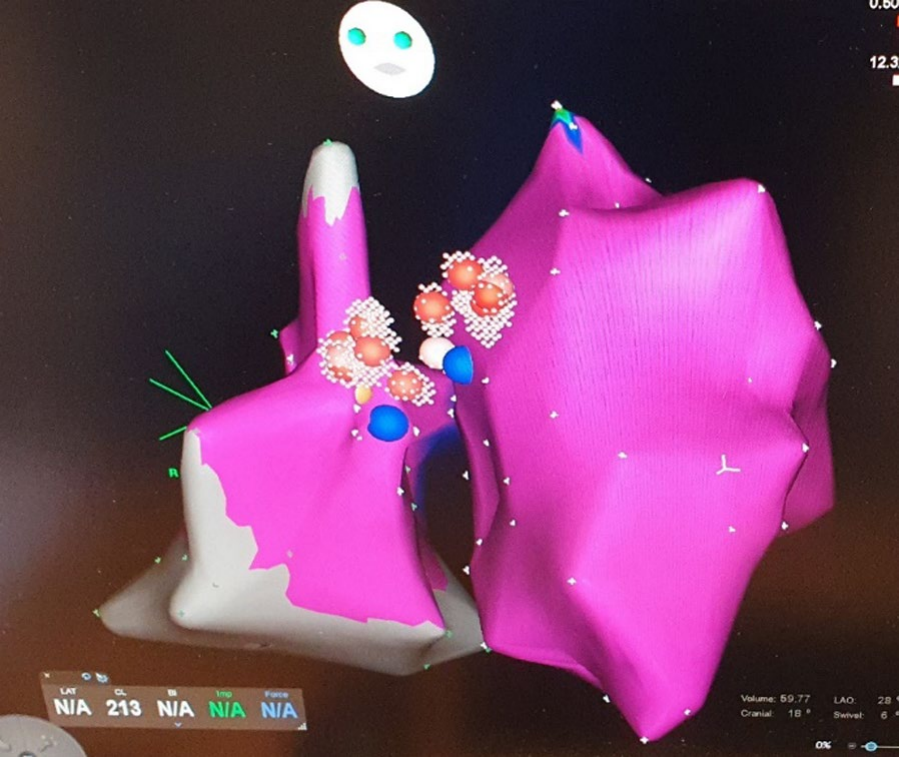
Radiofrequency catheter ablation lesions located bilaterally on the upper ventricular septum

In the following days, the episodes of BBRVT increased both in frequency and duration and progressed to ventricular storm, alternating between a RBBB and LBBB configuration (Fig. 1a, b). Lidocaine in addition to amiodarone had no effect on the tachycardia. Following a normal coronary arteriography, a repeated EP-study now revealed induction of two types of BBRVT, with LBBB and RBBB configurations corresponding to the 2 clinical VT’s. Voltage-mapping of both the right and left ventricles did not show signs of “myocardial scars”. VT-1 had local reentry-exit at the right sided basal part of the interventricular septum close to the RBB of the Hiss-Purkinje system. VT-2 had local VT-exit on the opposite part of the septum close to the LBB. Ablation was therefore performed to both sides of the basal ventricular septum after which the VT was non-inducible (Fig. 3). The heart rhythm now alternated between AFib and sinus rhythm with a markedly prolonged AV conduction time and a permanent RBBB pattern. An ICD was implanted during hospitalization.

Following supplemental prednisolone treatment, the patient then quickly recovered without shortness of breath, arrhythmia and with a normalization of both hemoglobin and CRP. Due to recurrence of symptoms and an episode af nonsustained ventricular tachycardia after prednisolone tapering, long-term treatment with prednisolone and mycophenolate mofetil were initiated without any relapse in the following 14 months. A control-CT-scan showed a marked reduction in aortic inflammation.

## Discussion

A bundle branch reentry ventricular tachycardia (BBRVT) is a rare form of VT that incorporates in its reentry mechanism both branches of the His-Purkinje system [8, 9]. The arrhythmia is most seen in patients with structural heart disease and concurrent significant deterioration of impulse propagation in the conduction system of the heart, such as patients with ischemic or dilated cardiomyopathy, arrhythmic right ventricular dysplasia or left ventricular non-compaction [8–10]. In contrast, neither acute bacterial endocarditis nor fulminant myocarditis appear to be associated with reentry-mediated VT, although case reports have been published of BBRVT in patients with Chagas disease [11].

In some patients infected by SARS-CoV-2, severe extra-pulmonary inflammation may develop without any preceding severe respiratory illness [12]. As such, initial reports of a new multisystem inflammatory syndrome in children (MIS-C) or in adults (MIS-A) as part of COVID-19 have surfaced. The syndrome denotes a severe dysfunction of one or more extra-pulmonary organ systems with concomitant laboratory evidence of inflammation following SARS-CoV-2 infection, but without severe respiratory illness [4]. MIS-A is described in patients approximately 2–5 weeks after COVID-19 infection, with generally mild cardiac signs and symptoms if present [12]. Only rarely is aortitis found to be related to a previous episode of COVID-19 [4, 5].

**Fig. 4:**
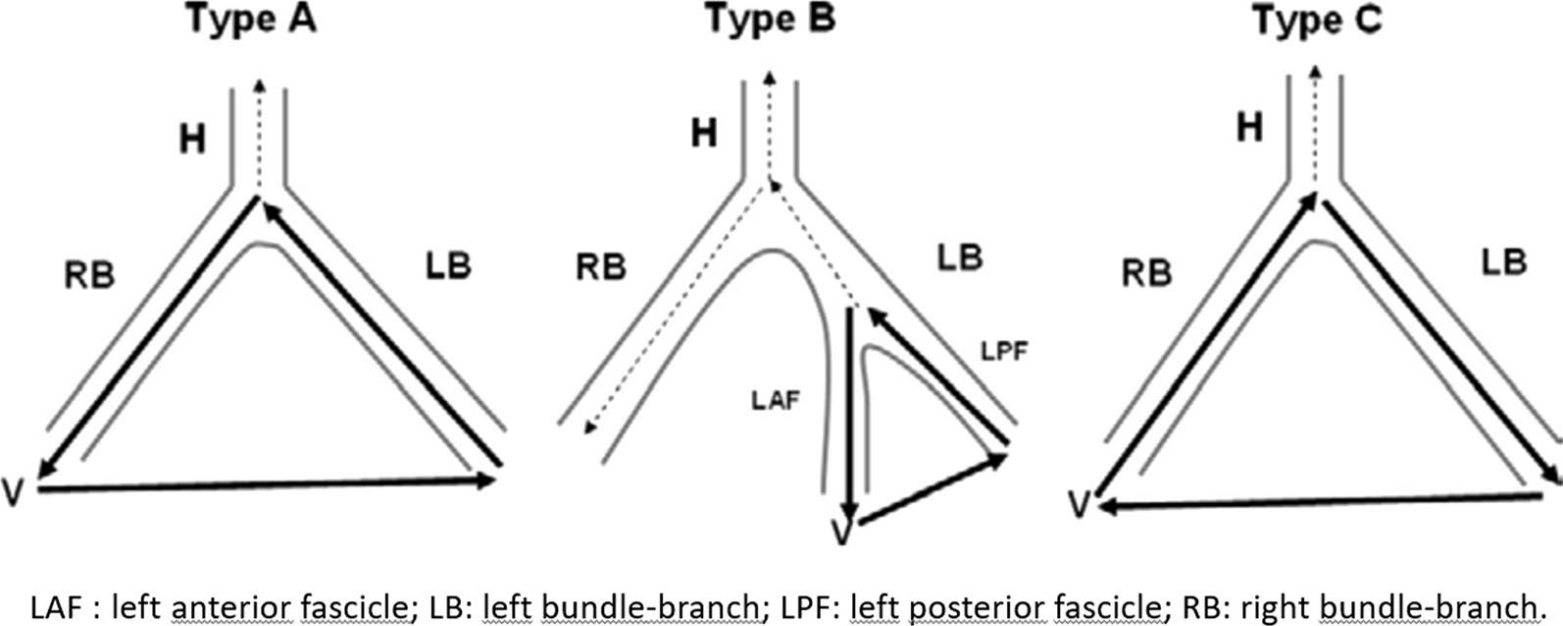
A schematic diagram showing the circuits in 3 types of bundle branch reentry

In the present case, a seemingly intractable BBRVT preceded by SARS-CoV2 infection was only managed following the diagnosis and management of aortitis. As with the present case, patients with BBRVT typically present with near-syncope and/or syncope. The resting ECG during sinus rhythm exhibits slightly widened QRS complexes, whereas a bundle branch block pattern is typically seen during VT, most often a LBBB pattern. Not all BBRVT are adenosine resistant [13]. Thus, the final diagnosis was established via an EP-study. In the present case a very fast initial deflection of the QRS of the surface ECG suggests exit of the VT close to the His-Purkinje System. BBR-VT originates from the His-Purkinje System and are most commonly associated with LBBB morphology but can be with RBBB – depending on the direction of the re-entry circuit (Fig. 4 – Type A and C), A fascicular tachycardias was unlikely, since it is bound to a specific and smaller part of the conduction system (i.e. fascicle; Fig. 4 Type B), which means it will not change in morphology from LBBB to RBBB as in the present case. Aberrant conduction of a SVT was unlikely to, because it requires a 1:1 AV conduction with up to 200 bpm with an RBBB morphology – which is unexpectedly fast considering the impaired and intermittently absent AV conduction. Finally, the EP study revealed a clear AV dissociation during both the LBBB and the RBBB tachycardias. RFA was considered first line therapy of BBR-VT and was performed with success in the present case. A subsequent need for permanent pacemaker therapy has been reported in up to 30% of RFA-treated BBRVT patients, due to damage to the impaired conduction system, with implantation of an ICD recommended as per standard guidelines [8]. In the present case ventricular function normalized during follow-up, but the history of ventricular storm and post ablation AV block indicated device implantation.

Given that the patient was known with a good AV conduction prior to COVID-19 infection and that no alternate etiology was established for the development of aortitis, the present case points at COVID-19 was the primary cause for the development of BBRVT. As COVID-19-related vasculitis also affects the surrounding tissues, it could be contended that the inflammatory response secondary to COVID-19 infection in the aortic root and thus presumably in the basal part of the ventricular septum led to AV block, thus enabling the substrate for the development of BBRVT. The role of aortitis in the development of BBRVT is further highlighted by the necessity as well as efficacy of prolonged steroid treatment following RFA. This case emphasizes that treatment targeting the underlying disease is mandatory and as such long-term anti-inflammatory treatment is necessary to prevent relapse and assure arrhythmia control in aortitis with myocardial affection.

An IgG4-related vasculitis may also affect the surrounding tissues causing a periarteritis like the aortitis presented in the actual case, but the IgG4 vasculitides are prone to the development of aneurysms. The serum IgG4-concentration was slightly elevated before prednisolone treatment was started, but the reliability of slightly elevated levels of the serum IgG4 concentration as a marker for the diagnoses of IgG4 aortitis has been questioned in the literature. Recently a case has been published showing regression after treatment with immunosuppression therapy with corticosteroid and mycophenolate mofetil of an IgG4 aortitis with diffuse peri-aortic thickening and aneurysmal dilatation also involving the coronary arteries [14]. Inherently we do not have the exact causal relationship between the previous COVID-19 infection and the inflammation of the aortic root and the basal part of the ventricular septum in the present case, but with a Covid-19 epidemic in the society and a recent COVID-19 infection, the possibility of a connection seems imminent.

## Conclusion

Aortitis is a rare, insidious and serious disorder, which is often discovered by chance. In the present case, the aortitis was preceded by a mild COVID-19 infection and discovered during the management of bundle branch reentry ventricular tachycardia – an in itself rare and life-threatening form of ventricular tachycardia. The present case is consistent with the recent recognition of a rare multisystem inflammatory syndrome in adults (MIS-A) following COVID-19 infection. As such, the present case is the first to describe a suspected COVID-19-related BBRVT. First choice of treatment for BBRVT is radiofrequency ablation, but treatment targeting the underlying disease is mandatory.

## Data Availability

Data and materials regarding this case are subject to National Danish Healthcare System Regulations but part of it can be sent in anonymized form by request to the corresponding author.

## Abbreviations

AFib: atrial fibrillation
AV: atrioventricular
BBRVT: bundle branch reentry ventricular tachycardia
EP-study: electrophysiological study
LBBB: left bundle branch block
MIS-A: multisystem inflammatory syndrome in adults
PCR test: Polymerase Chain Reaction (Covid 19) test
PET: positron emission tomography
RBBB: right bundle branch block
RFA: radiofrequency catheter ablation
VT: ventricular tachycardia
SVT: supraventricular tachycardia
